# Screening for Victims of Sex Trafficking in the Emergency Department: A Pilot Program

**DOI:** 10.5811/westjem.2017.2.31924

**Published:** 2017-04-17

**Authors:** Bryn E. Mumma, Marisa E. Scofield, Lydia P. Mendoza, Yalda Toofan, Justin Youngyunpipatkul, Bryan Hernandez

**Affiliations:** University of California, Davis, Department of Emergency Medicine, Sacramento, California

## Abstract

**Introduction:**

Estimates suggest that hundreds of thousands of sex trafficking victims live in the United States. Several screening tools for healthcare professionals to identify sex trafficking victims have been proposed, but the effectiveness of these tools in the emergency department (ED) remains unclear. Our primary objective in this study was to evaluate the feasibility of a screening survey to identify adult victims of sex trafficking in the ED. We also compared the sensitivity of emergency physician concern and a screening survey for identifying sex trafficking victims in the ED and determined the most effective question(s) for identifying adult victims of sex trafficking.

**Methods:**

We enrolled a convenience sample of medically stable female ED patients, age 18–40 years. Patients completed a 14-question survey. Physician concern for sex trafficking was documented prior to informing the physician of the survey results. A “yes” answer to any question or physician concern was considered a positive screen, and the patient was offered social work consultation. We defined a “true positive” as a patient admission for or social work documentation of sex trafficking. Demographic and clinical information were collected from the electronic medical record.

**Results:**

We enrolled 143 patients, and of those 39 (27%, 95% confidence interval [CI] [20%–35%]) screened positive, including 10 (25%, 95% CI [13%–41%]) ultimately identified as victims of sex trafficking. Sensitivity of the screening survey (100%, 95% CI [74%–100%]) was better than physician concern (40%, 95% CI [12%–74%]) for identifying victims of sex trafficking, difference 60%, 95% CI [30%–90%]. Physician specificity (91%, 95% CI [85%–95%]), however, was slightly better than the screening survey (78%, 95% CI [70%–85%]), difference 13%, 95% CI [4%–21%]. All 10 (100%, 95%CI [74%–100%]) “true positive” cases answered “yes” to the screening question regarding abuse.

**Conclusion:**

Identifying adult victims of sex trafficking in the ED is feasible. A screening survey appears to have greater sensitivity than physician concern, and a single screening question may be sufficient to identify all adult victims of sex trafficking in the ED.

## INTRODUCTION

Sex trafficking is defined as “recruitment, harboring, transportation, provision, obtaining, patronizing, or soliciting of a person for the purpose of a commercial sex act…” by “force, fraud, or coercion.”^1^,^2^ Hundreds of thousands of victims of sex trafficking and labor trafficking are estimated to exist in the United States,^3^ although accurate identification of victims is difficult due to the clandestine nature of trafficking. In 2015, 979 cases of human trafficking in California were reported to the National Human Trafficking Resource Center.^4^ While sex trafficking affects both women and men of all ages, the majority of victims are women^5^ with an average age at entry of 12–14 years old.^6^

Victims of sex trafficking have limited access to healthcare; any healthcare they receive frequently comes from the emergency department (ED). 7–9 Several “red flags” and questions for providers to identify victims of sex trafficking in healthcare settings have been suggested^10^–^13^ but are not well studied in the ED. The feasibility of using these screening questions in the ED and their ability to identify victims of sex trafficking in the ED are unknown.

Our objectives in this study were (1) to characterize the feasibility of using a screening survey to identify adult victims of sex trafficking in the ED, and (2) to compare a screening survey to physician concern for the identification of adult victims of sex trafficking in the ED.

## METHODS

### Study Design

We performed an observational cohort study in a single academic ED during a seven-month period from March to October 2015. We also surveyed treating emergency physicians regarding their concern for the patient being a victim of sex trafficking. Prior to the study, ED social workers were educated on sex trafficking and local resources available to victims. This study was approved by our institutional review board (IRB).

### Study Setting and Population

We surveyed a convenience sample of 143 female patients age 18–40 years in a single academic ED with an annual volume of 70,000 visits. Overall, 58% of the ED population is non-white. The surrounding county has a population of 1.5 million people, 64% of whom are white and 23% of whom are Hispanic or Latino. We selected women 18–40 years because they represent a substantial portion of the trafficked population. Furthermore, we were not able to enroll those less than 18 years of age because of the IRB requirement for informed consent. Prisoners and those in the custody of law enforcement were also excluded. Eligible patients were medically stable, able to provide informed consent, and able to read and understand either English or Spanish. Pregnant women were included. We surveyed patients at all times of the day.

### Screening Survey

As there are no validated screening tools for sex trafficking in the ED, we assembled a 14-question screening survey based on published recommendations,^10^–^13^ which could be administered in 5–10 minutes during the ED visit. We pilot-tested this survey on 15 ED patients. No significant changes were required after the pilot testing, and these patients were included in the overall study. Trained study personnel verbally administered the screening survey in a private ED treatment room without visitors present. A positive survey screen was defined as answering “yes” to any screening question(s).

### Emergency Physician Concern

During the ED visit, the treating ED resident or attending physician was asked whether they were “concerned that this patient may be a victim of sex trafficking.” This question was asked after the physician had completed his/her history and physical exam and prior to informing the physician of the survey screening result. Positive physician concern was defined as answering “yes” to this question.

### Social Work Consultation

All patients with positive screens or physician concern were offered social work consultation during their ED visit. ED social workers independently interviewed patients to understand their situations, assess their needs, and provide relevant resources.

### Data Collection and Management

Demographic and clinical data were abstracted from the EHR by trained study personnel. We managed study data using Research Electronic Data Capture (REDCap).^14^

### Outcomes

Our primary outcome was the feasibility of identifying victims of sex trafficking in the ED. Our secondary outcome was identifying a patient who was a victim of sex trafficking. We defined a “true positive” victim of sex trafficking as a patient acknowledgment of or social work documentation of sex trafficking.

### Analysis

We analyzed data using descriptive statistics with 95% confidence intervals (CI), where appropriate. Analyses were performed using Stata Version 14.1 (StataCorp LP, College Station, TX).

## RESULTS

We enrolled 143 women with median age 27 years (interquartile range 22–33 years) (Table 1). Overall, 46 patients screened positive for possible sex trafficking: 30 (21%, 95% CI [15%–29%]) on the screening survey only, seven (7%, 95% CI [2%–10%]) on physician concern only, and nine (6%, 95% CI [3%–12%]) on both. Ten (7%, 95% CI [3%–12%]) patients were confirmed victims of sex trafficking. None were identified by physician concern only.

All victims of sex trafficking listed the U.S. as their country of origin. The majority (80%, 95% CI [44%–97%]) had prior ED visit(s) within the prior two years, but only one (10%, 95% CI [2.5%–45%]) had visited a clinic within the study site’s health system. Victims presented to the ED with a broad range of chief complaints (Table 1).

Sensitivity of the screening survey (100%; 95% CI [70%–100%]) was better than physician concern (40%; 95% CI [12%–74%]) for identifying victims of sex trafficking, difference 60%; 95% CI [30%–90%]. Specificity of physician concern (91%; 95% CI [85%–95%]), however, was slightly better than the screening survey (78%; 95% CI [70%–85%]), difference 13%; 95%CI [4%–21%]. All (100%, 95%CI [74%–100%]) “true positive” cases answered “yes” to the following screening question: “Were you (or anyone you work with) ever beaten, hit, yelled at, raped, threatened or made to feel physical pain for working slowly or for trying to leave?” (Table 2).

## DISCUSSION

Identification of adult victims of sex trafficking in the ED using a brief screening survey is feasible. Our rate of “true positive” screens was surprising, particularly given prior reports that victims are often reluctant to disclose their situations in healthcare settings.^15^ Victims fear discrimination from healthcare providers, reporting to legal authorities, and punishment from their traffickers.^10^,^15^ The number of victims identified during this brief pilot study suggests that our ED regularly cares for victims of sex trafficking.

Consistent with other reports,^7^,^8^ victims in our study appeared to receive the majority of their healthcare in the ED. Our region has a high rate of sex trafficking,^5^ and it is likely that other EDs in our region also care for unrecognized victims. EDs are uniquely positioned to screen for sex trafficking and to provide interventions for victims. Victims of sex trafficking were not regularly recognized in our ED prior to this study. Thus, our study planning included research on available resources, community outreach, and emergency physician and social worker education. This multidisciplinary approach to caring for victims of sex trafficking, including physicians, nurses, social workers and community groups, is important for providing the ongoing support that these victims require. 9,10

One question was answered positively by all victims of sex trafficking (“Were you [or anyone you work with] ever beaten, hit, yelled at, raped, threatened or made to feel physical pain for working slowly or for trying to leave?”). This one question could be more easily incorporated into ED workflows than our 14-question screening survey. However, it remains unknown whether patients would answer this question positively if it were asked in isolation or whether the series of questions affects patients’ responses. Future research should evaluate the use of this question as a stand-alone screening tool.

Our screening survey had greater sensitivity than physician concern for identifying victims of sex trafficking. Several possible reasons exist. First, physicians may lack awareness of the risk factors for and signs of sex trafficking.^9^ Second, physicians may not ask questions about a patient’s social situation in the busy ED environment.^16^ In our study and others,^17^ victims presented with a variety of chief complaints that may not have prompted physicians to ask about their social situations or suspect them to be trafficking victims. Third, victims may hide their situation due to shame, fear, or distrust of the medical community.^16^ Victims’ traffickers may also be present, preventing them from disclosing their situation.^10^,^16^ The low sensitivity of physician concern makes it a less effective screening method than a screening survey. Future research should evaluate the effectiveness of physician- or nurse-administered screening question(s) integrated into patient care.

## LIMITATIONS

Our pilot study has several limitations. First, the “gold standard” for identifying victims of sex trafficking was patient acknowledgment or social work documentation of sex trafficking. It is possible victims of sex trafficking were never identified because they had a false negative screen or denied being victims following a positive screen. Second, our screening questions were derived from tools designed for other settings and had not been validated in an ED setting. Third, our study population for this small pilot study is a convenience sample from a single ED. Our results may not be generalizable to other settings, and a larger sample is required to draw definitive conclusions. Fourth, we were unable to obtain longer term follow-up on the effectiveness of our intervention for assisting victims of sex trafficking to escape their situation.

## CONCLUSION

Using a brief screening survey to identify of victims of sex trafficking in the ED is feasible. Our screening survey had greater sensitivity than physician concern, and a single screening question may be sufficient to identify all adult victims of sex trafficking in the ED.

## Figures and Tables

**Table 1:** Demographic and clinical characteristics of 143 emergency department patients in a study to determine the feasibility of identifying victims of sex trafficking.

	True Positives (n=10)	All other patients (n=133)
Demographic characteristics		
Age (years)	29±6	27±6
Race/ethnicity		
White	5 (50%)	41 (31%)
Black/African-American	3 (30%)	35 (26%)
Asian	0 (0%)	7 (5%)
Hawaiian/Pacific Islander	0 (0%)	3 (2%)
Native American/Alaskan	0 (0%)	2 (2%)
Hispanic/Latino	1 (10%)	26 (20%)
More than one race/ethnicity	1 (10%)	18 (14%)
Country of origin outside U.S.	0 (0%)	16 (12%)
Clinical characteristics		
Chief complaint		
Gynecological	3 (30%)	16 (12%)
GI/abdominal pain	2 (20%)	27 (20%)
Cardiac	0 (0%)	3 (2%)
Pulmonary	0 (0%)	8 (6%)
Neurologic	1 (10%)	15 (11%)
Trauma/injury	1 (10%)	37 (28%)
Substance use	1 (10%)	2 (2%)
Mental health	0 (0%)	2 (2%)
Other	2 (20%)	26 (20%)
ED visit(s) within 2 years	8 (80%)	71 (53%)
Clinic visit(s) in past 2 years	1 (10%)	42 (32%)

*US*, United States; *GI*, gastrointestinal; *ED*, emergency department.

**Table 2:** Questions and participant responses in a study to determine feasibility of using a brief screening survey to identify sex trafficking victims.

Screening tool items	“Yes” answers among true positive screens (n=10)	“Yes” answers among false positive screens (n=29)
Do you have to ask permission to eat, sleep, use the bathroom, or go to the doctor?	4 (40%)	2 (7%)
Were you (or anyone you work with) ever beaten, hit, yelled at, raped, threatened or made to feel physical pain for working slowly or for trying to leave?	10 (100%)	18 (62%)
Has anyone threatened your family?	6 (60%)	13 (45%)
Is anyone forcing you to do anything that you do not want to do?	5 (50%)	1 (3%)
Do you owe your employer money?	2 (20%)	1 (3%)
Does anyone force you to have sexual intercourse for your work?	5 (50%)	1 (3%)
Is someone else in control of your money?	4 (40%)	3 (10%)
Are you forced to work in your current job?	1 (10%)	0 (0%)
Does someone else control whether you can leave your house or not?	6 (60%)	1 (3%)
Are you kept from contacting your friends and/or family whenever you would like?	7 (70%)	6 (21%)
Is someone else in control of your identification documents, passports, birth certificate, and other personal papers?	4 (40%)	3 (10%)
Was someone else in control of arrangements for your travel to this country and your identification documents?	1 (10%)	0 (0%)
Do you owe money to someone for travel to this country?	0 (0%)	1 (3%)
Has anyone threatened you with deportation?	0 (0%)	1 (3%)
